# Supplementary material for: Quality of referral system and associated factors among referred clients referred to dessie comprehensive specialized hospital, Northeast, Ethiopia
Source: PLoS One. 2025 Dec 8;20(12):e0337715. doi: 10.1371/journal.pone.0337715 (PMC12685211; doi:10.1371/journal.pone.0337715)
Supplement: Attachment — Submitted filename: Response to Reviewers.docx [file pone.0337715.s003.docx]

Date: 01 /11/2024

Trhas Tadesse Berhe

PLOS ONE

Dear… D.r Trhas Tadesse Berhe

Thank you for giving us the opportunity to submit a revised draft of our manuscript titled

“Quality of Referral System and Associated Factors Among Referred Clients Referred to Dessie Comprehensive Specialized Hospital, Northeast, Ethiopia” to the PLOS ONE. We appreciate the time and effort that you and the reviewers have dedicated to providing your valuable feedback on our manuscript. We are grateful to the reviewers for their insightful comments on our paper. We have been able to incorporate changes to reflect most of the suggestions provided by the reviewers. We have highlighted the changes within the manuscript.

Please let us know if you still have any questions or concerns about the manuscript. We will be happy to address them, now in a timely manner.

Sincerely,

Yonas Fissha Adem

**Reviewer 1**

Reviewer #1: Dear author(s), thank you for your manuscript.
Overall, this is a clear, concise, and well-written manuscript on an important topic that is often under-researched. The introduction is relevant. Sufficient information about the importance of the research topic is presented for readers to follow the present study rationale and procedures. Although the article is well written but clarification of a few details should be provided.

**Response:** *dear reviewer, thank you very much for your motivational comment.*

Specific comments follow:
1. What was the reason for choosing the study method? As the study uses a cross-sectional design, Can this method establishes cause and effect relationships between variables?

**Response:** *dear reviewer, thank you for your valuable feedback.* a cross-sectional design *doesn’t show* cause and effect relationships between variables because it is collected data at a single point in a time. But before now there is not studies conduct in Ethiopian especially in the study areas so it better to know first prevalence of good quality of referral system. So, to achieved this purpose cross-sectional study was preferred to conduct.

2. What was the reason for choosing only one hospital? The study only includes patients referred to one hospital. This geographic limitation means the findings may not apply to other hospitals with different patient demographics or healthcare infrastructures.

**Response:** *dear reviewer, thank you for spotting this. We are choosing this hospital because it’s the only Comprehensive Specialized Hospital in wollo, which currently provides services for 10 million people in the South Wollo, North Wollo, Waghimra, and Oromia specialized zones.*

3. The article predominantly uses descriptive statistics to report results (e.g., frequencies and percentages), but lacks deeper inferential statistical analyses to explore more complex relationships between variables. This restricts the depth of insight that can be gained from the data.

**Response:** *Thank you for your thoughtful concern of our study. But this study was done both descriptive and analytical study. Descriptive study consists frequencies and percentages in this study. Analytical study consists comparison between dependent and independent variables using bivariable and multivariable binary logistic regression analysis in this study. So we consider this study have* deeper inferential statistical analyses.

4. While some operational definitions are provided, others like “good” and “poor” referral quality are based on arbitrary cutoffs (75%), without a robust explanation of why these thresholds were chosen. This raises questions about the validity and reliability of the quality assessments.

**Response:** *Thank you for your detailed comments. We are using “good” and “poor” referral quality system based on reference from previous studies. It isn’t arbitrary cutoffs. We have already putted a reference.*

5. Since data collection was done in a healthcare facility, patients might have provided more favorable responses due to social desirability bias, particularly in reporting their satisfaction. This bias could skew the findings, but the authors do not discuss how they managed or mitigated this issue.

**Response:** *Thank you so much for your careful check. But we have deployed four BSc. Nurses data collector and two BSc Nurses supervisor from other health facilities to reduce social desirability bias. The data collector informed the study participants that they came from other health facilities and its objective of the studies.*

6. The article mentions that verbal informed consent was obtained, but it does not explain why written consent was not sought or how verbal consent was documented, raising concerns about ethical rigor. Additionally, it does not address any potential risks to participants or how these were mitigated.

***Response:*** *Thank you for your valuable input.* *Verbal consent is appropriate for routine procedures that don't pose a significant risk to the patient and no need of written informed consent because we didn’t do any invasive procedures here rather we simply collected data from respondents. But we have incorporated the issue about mitigate* potential risks to participants.

7. Given the global trend towards electronic referral systems, the absence of any discussion on the potential role of digital solutions (e.g., e-referrals, telemedicine) in improving referral quality is a missed opportunity, especially in regions with infrastructure development plans like Ethiopia.

***Response:*** *Thank you for raising this important point. We have incorporated the issue you raised in the introduction part of the revised manuscript according to your comment.*

Reviewer #2: The main text mentions Table 7, but the table does not actually exist. Please recheck the arrangement and ensure that all referenced tables are correctly accounted for.
The resolution of the figures should be improved. Please resubmit new, clearer figures to ensure better publication quality.

***Response:*** *We would like to thank the dear reviewer for your frank comment. We reviewed and made adjustments in the revised manuscript.*

Conclusion subtitle has a unexpected dot (line 412)

***Response:*** *We appreciate your valuable suggestion. We have made corrected in the revised manuscript as per your expectations.*
