# Supplementary material for: Quality of referral system and associated factors among referred clients referred to dessie comprehensive specialized hospital, Northeast, Ethiopia
Source: PLoS One. 2025 Dec 8;20(12):e0337715. doi: 10.1371/journal.pone.0337715 (PMC12685211; doi:10.1371/journal.pone.0337715)
Supplement: Attachment — Submitted filename: Response_to_Reviewers_auresp_2.docx [file pone.0337715.s004.docx]

Date: 01 /06/2025

Trhas Tadesse Berhe

PLOS ONE

Dear… D.r Trhas Tadesse Berhe

Thank you for giving us the opportunity to submit a revised draft of our manuscript titled

“Quality of Referral System and Associated Factors Among Referred Clients Referred to Dessie Comprehensive Specialized Hospital, Northeast, Ethiopia” to the PLOS ONE. We appreciate the time and effort that you and the reviewers have dedicated to providing your valuable feedback on our manuscript. We are grateful to the reviewers for their insightful comments on our paper. We have been able to incorporate changes to reflect most of the suggestions provided by the reviewers. We have highlighted the changes within the manuscript.

Please let us know if you still have any questions or concerns about the manuscript. We will be happy to address them, now in a timely manner.

Sincerely,

Yonas Fissha Adem

**Point by Point Response to - Editor**

**Point by Point Response to – Reviewer**

Reviewer #3: Thank you for the opportunity to review this manuscript. It is a worthwhile study of an interesting subject; however there are a number of things that should be addressed before it is published.

The first 2 sentences of the abstract are not relevant to the study and should be deleted.
***Response:*** *We appreciate your thoughtful observation regarding this issue. We've made changes to this section based on your comments.*

The components assessed to determine the overall quality, input quality and process quality of the referral are not provided until the Tables in the body of the results. It would be helpful to have some examples in the Abstract and Methods eg Process Quality measured whether the important patient demographic and clinical information was included in the referral, Outcome quality was based on the patient’s impression of their referral etc.
***Response:*** *Thank you for your suggestion, dear. However, in this study, the components of the quality referral system (structure, process, and outcome) have already been mentioned in the operational definition section of the methods. We can explain their meanings in the abstract, but to keep it short and precise; we haven’t mentioned them in the abstract.*

It is not clear what “focal person” means. Does it mean ‘contact person’? The presence or absence of a “focal person” is mentioned in the introduction and discussion, but not in the methods or results so it is not clear how this was measured or what was found. Either the methods and results should be included, or the concept removed from the introduction and discussion.
***Response:*** *We appreciate your thoughtful observation regarding this issue. But, We've already incorporated about “focal person” in the Method part “operational definition” and in result part “Structural (input) part of the referral system”. "****Focal person****" typically refers to an individual designated to oversee and coordinate activities related to a specific program, initiative, or area of focus within the hospital.*

The recruitment strategy and numbers do not seem realistic and require clarification. The methods state that every 4th patient of an expected 2000 patients per month was approached and interviewed. It is inconceivable that there were no protocol violations during this process. What happened if patients presented overnight, if multiple patients presented at once, if patients were too confused or unwell or not available to respond to questions, if the research staff were sick? The actual recruitment strategy should be explained. If it is a convenience sample of ~1/4 of patients presenting to the hospital, that should be stated.

***Response:*** *Thank you for your suggestion, dear. However, this study's recruitment strategy was systematic random sampling, selecting every 4th patient out of an expected 2,007 patients monthly. Multiple patients cannot be presented at once because their charts are organized based on their assigned order. Therefore, every 4th patient was selected; patients can come during the night, but patient chart was given based on the order so no problem for random patient selection, but the data collection was conducted in day. If a patient was confused or unable to communicate, the next random sample was taken. No research staffs were sick during our study, but if any staff member had fallen ill, other data collectors would have been deployed.*

Reference 16 reports that in Tigray, 82% of patients were self-referred. Dessie Comprehensive Specialized Hospital presumably treats similar numbers of self-referred patients. Are the 2000 patients per month only the ‘referred’ patients, (ie does the hospital see 10000 patients per month, of whom 20% are referred) or is this all the patients, and what proportion were referred? How did the research staff distinguish between self-referred patients and those transferred from other hospitals on foot or by public taxi, some of whom are likely to have lost their referral papers? Inclusion or exclusion of patients with missing papers could significantly change the results.
***Response:*** *Thank you for your suggestion dear, but this study is aimed to assess the quality of referral system but not about magnitude of self-referred patients. Every patient should bring referral paper so if it’s lost it should be rewrite again to get treatment from Hospital since its referral hospital. But in our stud, no patients loss the referral paper. So no need of putting this idea in the inclusion and exclusion criteria.*

Patient satisfaction is reported as 44.6% overall, but the individual questions suggest a much higher level of satisfaction. How was the 44.6% overall satisfaction derived? Similarly, the overall quality of the referral system is reported as 62.5%, but in the data tables 94.7% of the referrals were good, and 87.3% of the feedback was good. Can this please be clarified? Similarly, the conclusions that’ generally clients had poor referral quality’ are not supported by the data, which shows that most referrals were good quality.
***Response:*** *Thank you for your suggestions. In this stud, the overall patient satisfaction with the referral system of health facilities to Dessie Comprehensive Specialized Hospital was found to be 44.6%, this result was found by calculating the average value of each 8 measuring variables of patient satisfaction for each individual then patients who scored above the half(50%) was considered satisfied. Even when you see each individual variables value, it’s not highest and it was classified into five. In addition to this the overall quality of referral system was computed by three components of quality. So even if the structural and process parts of the referral system have highest score which was 90.3% and 94.7% respectively. But the outcome (patient satisfaction) was poor which score 44.6%. So the overall quality of referral was calculated by computing the above three referral system so final we have got 62.5% of the patients scored 75 % and above.*

The paper would benefit from editing to improve the academic English.

***Response:*** *Thank you for your suggestions. We will improve the whole document based on your comments.*
