# Supplementary material for: Quality of referral system and associated factors among referred clients referred to dessie comprehensive specialized hospital, Northeast, Ethiopia
Source: PLoS One. 2025 Dec 8;20(12):e0337715. doi: 10.1371/journal.pone.0337715 (PMC12685211; doi:10.1371/journal.pone.0337715)
Supplement: Attachment — Submitted filename: Response to Reviewers 3.docx [file pone.0337715.s005.docx]

Date: 20 /08/2025

Trhas Tadesse Berhe

PLOS ONE

Dear… D.r Trhas Tadesse Berhe

Thank you for giving us the opportunity to submit a revised draft of our manuscript titled

“Quality of Referral System and Associated Factors Among Referred Clients Referred to Dessie Comprehensive Specialized Hospital, Northeast, Ethiopia” to the PLOS ONE. We appreciate the time and effort that you and the reviewers have dedicated to providing your valuable feedback on our manuscript. We are grateful to the reviewers for their insightful comments on our paper. We have been able to incorporate changes to reflect most of the suggestions provided by the reviewers. We have highlighted the changes within the manuscript.

Please let us know if you still have any questions or concerns about the manuscript. We will be happy to address them, now in a timely manner.

Sincerely,

Yonas Fissha Adem

**Point by Point Response to - Editor**

**Point by Point Response to – Reviewer**

Thank you for your response. However, the explanation regarding the feasibility of systematic sampling and the absence of any protocol violations appears unrealistic. Please revise your response and the Methods section to acknowledge potential real-world challenges (e.g., patient unavailability, staff absence, night-time arrivals), and explain how such issues were handled or mitigated. Transparency about possible limitations is important for methodological rigor.

***Response:*** *Response: We appreciate your thoughtful observation regarding this issue. We've made changes to this section based on your comments.*

-the reviewer’s request was not fully addressed. While the components of quality (structure, process, and outcome) are defined in the operational definitions, the reviewer specifically asked for brief examples to be included in the Abstract and Methods sections for clarity.Please revise the Abstract to include a short example for each component, and add a concise explanation in the Methods section to improve reader understanding

***Response:*** *Thank you for your suggestions.* *We've made incorporated to the Abstract and Methods section based on your comments.*

-While the term “focal person” is defined in the operational definitions and mentioned in the results, it would benefit readers if it were clearly introduced and defined upon first use in the Introduction. Please revise the manuscript to define the term earlier in the narrative to improve clarity and flow.

***Response:*** *Thank you for your suggestions. We have defined the raised issue in the introduction part.*

-the reviewer’s concern was not fully addressed. While we understand the focus was not on self-referral, the issue raised relates to potential sample bias due to possible exclusion of patients who may have lost referral papers.

Please clarify how such cases were handled and revise the inclusion criteria and limitations sections to reflect this, ensuring transparency in your methodology.

***Response:*** *Thank you for your suggestions.* *But in our study, no patients loss the referral paper. We want to ask a great apologize for making a mistake here and we have replaced loss the referral paper with unable to communicate in the exclusion criteria.*

-The explanation regarding the overall satisfaction score and referral quality remains unclear. There appears to be a misalignment between the findings and the conclusion, particularly as structure and process scores were high, yet the conclusion states that clients experienced “poor referral quality.”

Please provide a clearer explanation of how the overall quality score was calculated, especially how satisfaction was incorporated. Also, revise the conclusion to more accurately reflect the mixed findings  for example, acknowledging high structural and process performance alongside lower patient satisfaction.

***Response:*** *Thank you for your suggestions. The perceived misalignment arises because the overall quality of the referral system was calculated by considering three components of quality measures: input, process, and outcome. While the structural (input) and process aspects scored highly, the outcome component, specifically patient satisfaction, was considerably lower, which pulled down the overall quality score.*

*•* ***Structural (Input) Quality:*** *The overall structural input quality of the referral system was* ***90.3%****. This indicates a high availability of essential components like a referral focal person, registry book, standard referral paper, and transport.*

*•* ***Process Quality:*** *The overall quality related to the referral and feedback papers was also high.*

*◦ The overall quality of the* ***referral paper was good in 94.7%*** *of participants.*

*◦ The score of components of* ***feedback sent was good in quality in 87.3%*** *of study subjects, and the* ***overall quality of the referral feedback was good in 87.7%*** *of participants.*

*◦ The* ***overall process quality was good in 88.3%*** *of study participants.*

*•* ***Outcome (Patient Satisfaction):*** *This is where a significant difference is observed. The* ***overall patient satisfaction*** *with the referral system at Dessie Comprehensive Specialized Hospital was only* ***44.6%*** *(95% CI: 40.00%–49.20%). For example, only 68.3% of referred patients agreed that health professionals explained the referral in a way they could understand, and 62.5% agreed that professionals included them in deciding the reason for the referral.*

*•* ***Overall Referral Quality:*** *When all three components (input, process, and outcome) were considered, the* ***overall quality of the referral system was computed to be 62.5%*** *(95% CI 57.6%-66.8%), meaning* ***37.5% was considered poor in quality****.*

- *The conclusion part has accordingly modified*

-Thank you for acknowledging the need to improve the language quality of the manuscript. However, your response does not indicate whether a thorough language revision has been carried out.

To ensure clarity and academic quality, please revise the manuscript with support from a native English speaker or a professional language editor, and submit a track-changed version highlighting the edits made.

***Response:*** *Thank you for your suggestions. We have improved as much as possible. But we didn’t highlight the change because many grammatical and spelling correction might presented.*
