# Supplementary material for: Quality of referral system and associated factors among referred clients referred to dessie comprehensive specialized hospital, Northeast, Ethiopia
Source: PLoS One. 2025 Dec 8;20(12):e0337715. doi: 10.1371/journal.pone.0337715 (PMC12685211; doi:10.1371/journal.pone.0337715)
Supplement: Attachment — Submitted filename: Response to Reviewers 4.docx [file pone.0337715.s006.docx]

Date: 01 /10/2025

Trhas Tadesse Berhe

PLOS ONE

Dear… D.r Trhas Tadesse Berhe

Thank you for giving us the opportunity to submit a revised draft of our manuscript titled

“Quality of Referral System and Associated Factors Among Referred Clients Referred to Dessie Comprehensive Specialized Hospital, Northeast, Ethiopia” to the PLOS ONE. We appreciate the time and effort that you and the reviewers have dedicated to providing your valuable feedback on our manuscript. We are grateful to the reviewers for their insightful comments on our paper. We have been able to incorporate changes to reflect most of the suggestions provided by the reviewers. We have highlighted the changes within the manuscript.

Please let us know if you still have any questions or concerns about the manuscript. We will be happy to address them, now in a timely manner.

Sincerely,

Yonas Fissha Adem

**Point by Point Response to - Editor**

**Point by Point Response to – Reviewer**

**Systematic Sampling & Real-World Challenges**
while your revision briefly mentions night-time arrivals and uncommunicative patients, the explanation remains limited. Kindly expand the *Methods* section to explicitly acknowledge common real-world challenges (e.g., patient unavailability, staff absence, timing of arrivals) and describe how these were handled in practice.

***Response:*** *We appreciate your thoughtful observation regarding this issue. We've made changes to this section based on your comments in the sampling procedures and technique.*

**Examples of Quality Components (Abstract and Methods)**
In the *Methods* section, you have outlined structure, process, and outcome components. However, the *Abstract* still does not include short illustrative examples (e.g., structure = referral forms/focal person, process = referral and feedback papers, outcome = patient satisfaction). Kindly revise the *Abstract* accordingly for clarity.

***Response:*** *Thank you for your suggestions.* *We've made incorporated this issue in the Abstract section previously but we have put it short and precisely again based on your comments.*

**Sample Bias Referral Papers**
The reviewer raised concerns about potential bias if patients lost referral papers. Your current response simply replaces this with “unable to communicate” in the exclusion criteria. Please clarify whether any such cases occurred, how they were handled, and acknowledge this as a possible limitation if applicable.

***Response:*** *Thank you for your suggestions.* *But in our study, no patient’s lost the referral paper in reality. But we have incorporated this issue in the sampling procedures and technique part* *based on your comments. If we have got patient lost referral paper the next random sample were taken.*

**Language Revision**
Although you mention improving the manuscript, there is no evidence that a full language revision was carried out. Given the number of grammatical and stylistic issues still present, we strongly recommend that the manuscript be thoroughly revised by a native English speaker or a professional editor before resubmission.

***Response:*** *Thank you for your suggestions. We have improved as much as we can. But we didn’t highlight the change because many grammatical and spelling corrections might presented.*
